# Genetic Pathways Associated With Sleep Problems in Children With Autism Spectrum Disorder

**DOI:** 10.3389/fpsyt.2022.904091

**Published:** 2022-07-08

**Authors:** Ping-I Lin, Anne Masi, Mohammad Ali Moni, Sarah Kummerfeld, Valsamma Eapen

**Affiliations:** ^1^School of Psychiatry, Faculty of Medicine, University of New South Wales, Sydney, NSW, Australia; ^2^Mental Health Research Unit, South Western Sydney Local Health District and Ingham Institute, Sydney, NSW, Australia; ^3^Academic Unit of Psychiatry, Infant Child and Adolescent Mental Health Services, South Western Sydney Local Health District and Ingham Institute, Sydney, NSW, Australia; ^4^Artificial Intelligence and Digital Health Data Science, School of Health and Rehabilitation Sciences, Faculty of Health and Behavioural Sciences, The University of Queensland, St. Lucia, QLD, Australia; ^5^Kinghorn Centre for Clinical Genomics, Garvan Institute of Medical Research, Sydney, NSW, Australia; ^6^St. Vincent's Clinical School, University of New South Wales, Sydney, NSW, Australia; ^7^Cooperative Research Centre for Living With Autism (Autism CRC), The University of Queensland, Indooroopilly, QLD, Australia

**Keywords:** autism spectrum disorder, sleep disturbance, melatonin, genomewide association study, pathway analysis

## Abstract

**Aims:**

Children on the autism spectrum are more likely to have sleep problems than non-autistic children. Sleep disturbance may exacerbate emotional and behavioral problems of children on the autism spectrum. A better understanding of the biological mechanisms underlying sleep disturbance provide clues to better management for this co-morbid condition in autism. The goal of the current study is to identify genetic variants associated with sleep disturbance and melatonin levels in autistic children.

**Methods:**

A total of 969 children on the autism spectrum were genotyped using the Global Screening Array v1 or Global Screening Array v2. Sleep problems were assessed using the Children's Sleep Habits Questionnaire (CSHQ). Melatonin levels were measured using the urine samples of 219 probands. The relationship between the melatonin level and CSHQ score was examined using the general linear model. The genetic variants associated with the CSHQ score and melatonin level as two separate quantitative traits were determined using genomewide association studies.

**Results:**

The data indicates that urine melatonin levels were positively associated with CSHQ scores, suggesting that autistic children with a poorer sleep qualiy could has higher melatonin level. Furthermore, genetic assocication studies suggest that genetic pathways involved in pro-inflammatory responses might be involved in sleep disturbance, while genetic pathways involved in catecholamine-secreting PC12 cells and Schwann cells could be associated with melatonin levels.

**Conclusions:**

Taken together, our findings indicate that sleep disturbance and melatonin metabolism could be attributable to distinct biological mechanisms in autistic children since they might not share genetic contributors.

## Introduction

Extensive evidence has indicated the link between sleep disturbance and mental health issues. Children on the autism spectrum and their families and caregivers are already at high risk of poor mental health, such as depressed mood and anxiety. A comorbid sleep disorder is likely to compound these risks and significantly impact the quality of life and functioning of the whole family. The debilitating nature of sleep disturbances and disordered sleep patterns can have a profound effect on quality of life, wellbeing and stress levels of both child on the autism spectrum and their families (1).

The incidence rate of sleep disturbances, such as sleep latency, waking after sleep onset, and shorter sleep duration, of children on the autism spectrum is ~4 times higher than the incidence rate in non-autistic children (2). The association between the severity of sleep problems with the level of autism traits has been widely reported. Specifically, lower sleep duration has been associated with high level of core autism traits, and sleep duration and IQ scores were found to be positively correlated. Furthermore, compromised sleep is related to an exacerbation of problematic daytime behaviors. Given these findings, sleep has emerged as one of the most clinically impairing cooccurring conditions for children on the autism spectrum and the profound adverse impact on the families and their wellbeing and functioning highlights its importance as a research priority.

Given the prevalence of sleep problems in this population it is imperative that a deeper, more comprehensive understanding of the contributing factors are elucidated. Although accumulating evidence indicates that sleep disturbance predicts emotional and behavioral problems (3–5) and physical health concerns (6, 7) in children on the autism spectrum, biological mechanisms underlying sleep problems in autism remain unclear. Melatonin is considered as the biomarker that is most strongly correlated with sleep quality among individuals with neurodevelopmental disorders (8). Lower levels of the major metabolite of melatonin, urinary 6-sulfatoxymelatonin (6-SM), have also been identified in autistic individuals (9). Furthermore, the the genes in the melatonin metabolism pathways—such as Acetylserotonin O-Methyltransferase (ASMT) gene that encode the last enzyme of melatonin synthesis—may contribute to sleep problems in individuals on the autism spectrum (10, 11). The identification of genetic variants associated with sleep disturbance and melatonin levels in individuals on the autism spectrum may cast some insights into biological predispositions to sleep disturbances in autism—which in turns can pave the way for novel therapeutic strategies.

To explore the role of genetic factors in sleep disturbances in autism, we attempted to address the following research questions:

1. What is the relationship between the melatonin level in the urine and sleep disturbances in children on the autism spectrum?
2. Which genetic variants are associated with sleep disturbances in children on the autism spectrum?
3. Which genetic variants are associated with the variation in melatonin levels in children on the autism spectrum?

## Materials and Methods

### Participants

All data were obtained from the Australian Autism Biobank (AAB) (12) provided by the Cooperative Research Center for Living with Autism (Autism CRC). Participants in this study were children diagnosed with autism spectrum disorder per DSM-IV or DSM-5 criteria, depending on their age at diagnosis. No exclusion criteria were applied with respect to conditions other than autism, such as other psychiatric, medical or genetic conditions, cognitive function level, or medication use.

### Measures for Key Variables

The Children's Sleep Habits Questionnaire (CSHQ) was used to assess sleep quality (13). Subscales of the CSHQ relevant to the research area on the effect of melatonin on autism; sleep latency (bedtime resistance), waking after sleep onset, sleep duration, and created a combined total score based on these three sub-sections. Each subscale had equal weight in the combined score.

Melatonin levels were measured using the urine samples of 219 probands were extracted from the AAB. Concentrations of the primary metabolite of melatonin-6-sulfatoxymelatonin—were determined using an enzyme-linked immunosorbent assay (ELISA). Blinded analysis of urine 6-sulphatoxymelatonin (6-SM) levels was performed by enzyme immunoassay using an assay kit from IBL International (Hamburg, Germany). The urine samples were clarified prior to testing. The intra–assay coefficient of variation was 11–15% (*n* = 12) for 14 and 72 ng/ml control samples, respectively. Excretion of 6-SM was expressed in ng/mL.

The DNA extracted from peripheral mononuclear blood cells for 2,489 subjects including probands on the autism spectrum, parents, and unaffected siblings from the AAB was used to analyse the single nucleotide polymorphisms (SNPs) across the whole genome. A total of 1,269 probands had the data on genotypes and CSHQ scores. Preparation for genotyping analysis included pre-imputation QC, ethnicity derivations, imputation (using ENIGMA genetics protocols) and post-imputation QC. The study was conducted in accordance with the declaration of Helsinki under the research protocol approved by the ethics committee of University of New South Wales, Australia.

### Statistical Data Analyses

#### Descriptive Statistics

The distributions of the data on demographics (i.e., age and gender), sleep quality (indicated by the total CSHQ scores), and 6-SM levels, were shown using frequency values for categorical variables and mean/SD for continuous variables. evaluated using R version 3.6.3 (R Core Team, 2020). An R data library was created for the CSHQ and autism clinical scores using the devtools library.

#### Melatonin Analysis

The total CSHQ scores were used to measure the level of sleep quality, which can be used to identify individuals with sleep disturbances. Since 6-SM levels were measured using the urine samples from the participants were collected in two different times of the day (5.00 a.m. to 11.55 a.m. and 12.00 p.m. to 9.02 p.m.) the batch effect of metabolite levels was corrected using the proportion of maximum scoring (POMS) or min-max scaling approach in rescaling all features to 0–1 range by applying the following transformation: (*x* and *z* represent original value and transformed value, respectively).

$$z_{i}=\frac{x_{i}-x_{min}}{x_{max}-x_{min}}$$

However, normalized 6-SM levels were still highly skewed, so the data on 6-SM levels were processed using the rank-based inverse normal transformation technique. To examine the relationship between 6-SM levels and total CSHQ scores, we used general linear model to regress transformed 6-SM levels against normalized 6-SM levels adjusting for the age, gender, and the level of autism traits (indicated by the ADOS comparison score). Additionally, since melatonin rhythms are altered in a variety of circadian rhythm, we also adjusted for the time of sample collection (i.e., morning vs. afternoon) in the general linear model. The two-sided alpha value of 0.05 was used to determine the level of statistical significance.

#### Genome-Wide Association Study

Two quantitative trait genome wide association studied (GWAS) were carried out to identify genetic variants associated with sleep quality (measured with the CSHQ scores) and the variation in 6-SM levels, respectively. Participants were genotyped using either the Global Screening Array v1 or Global Screening Array v2 (Illumina, San Diego, CA).

1. *Qualitative Control for genotype data*: SNPs with a genotyping frequency < 95%, Hardy-Weinberg Equilibrium (HWE) *P*-value < 1 × 10^−5^, or minor allele frequency (MAF) <0.1%, were eliminated from the GWAS. Additionally, sex discrepancy and relatedness (the default cutoff value for genomic relateness is 0.025) were examined using the PLINK [v1.90b5 64-bit (November 14, 2017)].
2. The quantitative association analysis analyses were carried out software package. ADOS scores were also adjusted in the models for both GWAS while and time of urine sample collection was adjusted for for the GWAS of 6-SM levels. Manhattan, Q-Q and *P*-value distribution plots were generated using the qqman pckage in R version 3.6.3. We consider that the traditional GWAS significance threshold, *P*-value < 5 × 10^−8^, might be too conservative because it controls type 1 errors (false-positives) at the price of inflating type 2 errors (false negatives) (14). Therefore, we implemented a less stringent criterion to determine putative genotype-phenotype associations by using 1 ×10^−5^ as a significance threshold.

#### Gene Set Analysis

We used an exploratory and less stringent significance threshold to select the top 0.01% of the SNPs as loci associated with the individual phenotype to identify genetic networks. Therefore, a total of 25 SNPs was selected from each phenotype. No trait-associated SNPs were shared by the two sets of loci associated with the sleep quality (i.e., CSHQ score) and 6-SM level, respectively. We found the *P*-value cut-off for sleep data was < 2.5 × 10^−5^) and the *P*-value cut-off for 6-SM data was <3.5 × 10^−5^. We have used the Ensemble Variant Effect Predictor (http://asia.ensembl.org/info/docs/tools/vep/index.html) to predict and identify the mapped genes. We then performed the gene set enrichment analysis of Gene Ontology and cell signaling pathways to evaluate the biological relevance and functional pathways of these mapped genes for both mapped gene lists. We have incorporated the KEGG (15), WikiPathways (16), BioCarta (17), and Reactome (18) pathway database for the cell signaling pathways. We have also considered the GO Biological Process database for gene ontological analysis (19). The GO terms and pathways enriched by the list of genes were identified using the hypergeometric analyses with an adjusted *P* ≤ 0.05 was considered as statistically significant.

## Results

### Characteristics of the Sample

A total of 2,489 subjects had genotypic data at ABA. The analyses used to evaluate the association between sleep problems and genotypes were only conducted for the subjects with available CSHQ scores (*N* = 969). The 6-SM levels were available in 22.6% of the probands (*N* = 219). The mean values of age, ADOS scores, and CSHQ scores, were 7.63 (SD: 3.86), 6.69 (SD: 1.92), and 47.92 (SD: 9.98), respectively. The sample of probands was predominantly males (78.74%).

### The Relationship Between the 6-SM Level and the CSHQ Score

**Figure 1:**
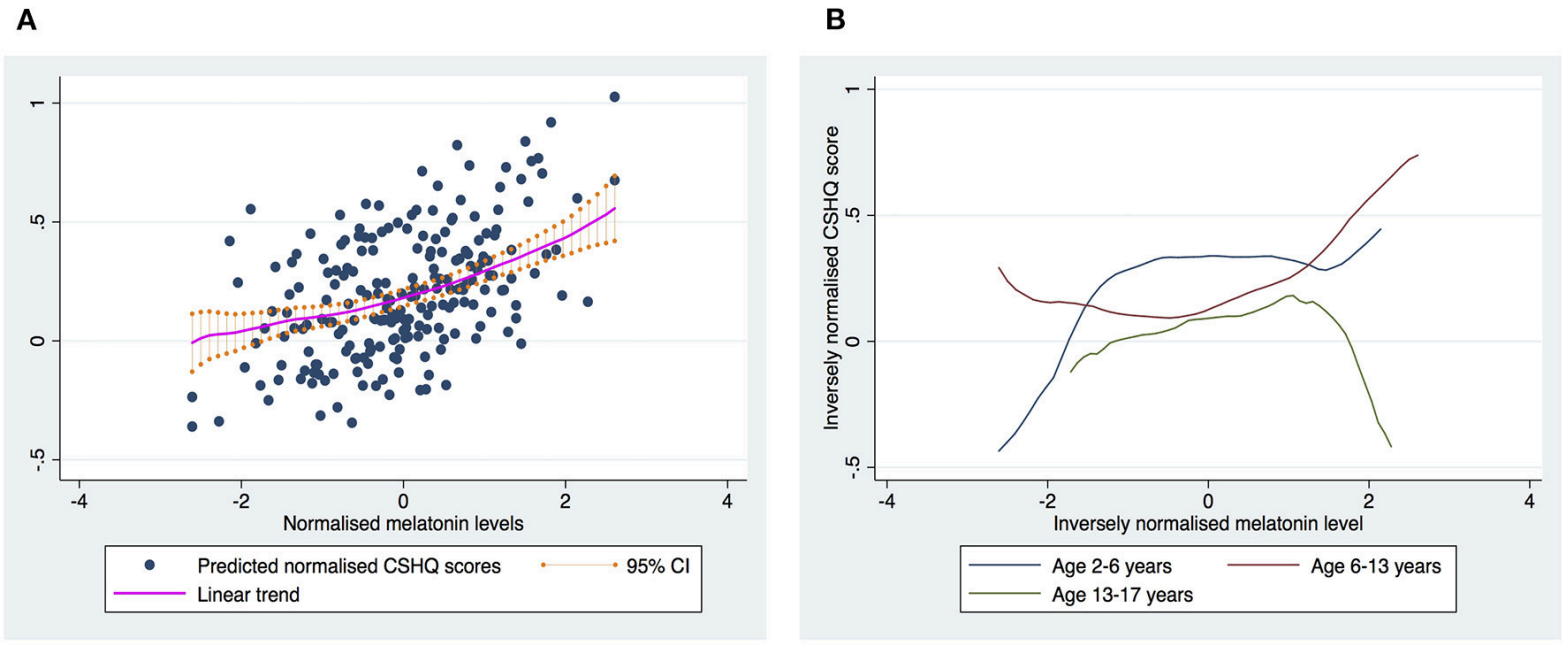
The relationship between melatonin levels and CSHQ score. **(A)** Shows the overall trend for the relationship between melatonin levels and CSHQ scores. **(B)** Shows the trends stratified into three age groups. The curves were based on the estimates using the Locally-Weighted Scatterplot Smoothing (LOWESS) regression model.

Normalized 6-SM levels were statistically significantly associated with the CSHQ scores (***p* =** **0.0371**) after adjusting for data collection time, gender, age, and ADOS2 comparison score. The linear relationship indicates that a higher 6-SM level was associated with a higher CSHQ total score (see Figure 1A).

Since melatonin levels vary by age, we further examined the relationship between CSHQ scores and 6-SM levels by age group, and found that the trend id vary by age (see Figure 1B). Therefore, the positive association between 6-SM levels and CSHQ scores seemed to be driven by younger children.

### Genetic Variants Associated With the Target Phenotypes

**Figure 2:**
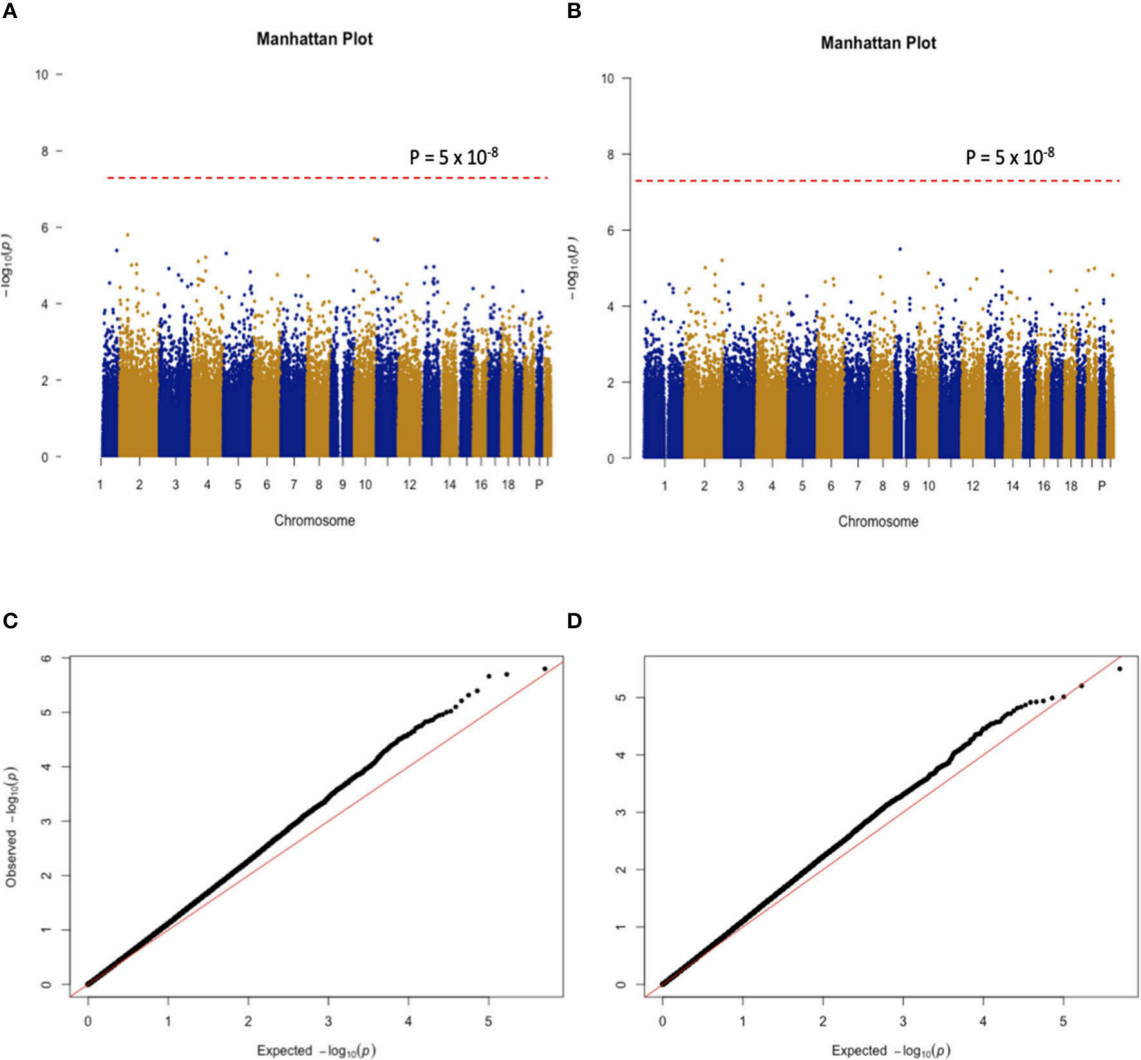
**(A,B)** Show the Manhattan plots for the GWAS findings with CSHQ scores and melatonin levels, respectively. **(C,D)** Only autosomal chromosome results are shown here.

When the CSHQ score was treated as a phenotype, the GWAS results indicate that none of the SNPs reached the stringent genome-wide significance level (*p* < 5 × 10^−8^). There was a total of nine SNPs with a *P*-value < 1 × 10^−5^. The SNP with the strongest evidence for association with the CSHQ score was rs13011288, which is located in the intronic region of the *Stonin 1* (STON1) gene. When the 6-SM level was treated as a phenotype, the GWAS results indicate that none of the SNPs reached the stringent genome-wide significance level (*p* < 5 × 10^−8^). The SNP with one of the strongest lines of evidence for its association with the 6-SM level is rs1543334 located ~10 kbp away from the *Homo Sapiens Synaptosome Associated Protein 25* (SNAP25) gen*e* (*p* = 1.15 × 10^−5^). Note that it could not be definitivey considered as the trait-associated locus according to the significance threshold in the context of GWAS. The Manhattan plots of the two GWAS studies are shown in Figure 2A, while Q-Q plots are shown in Figure 2B.

### Gene Set Analysis

**Figure 3:**
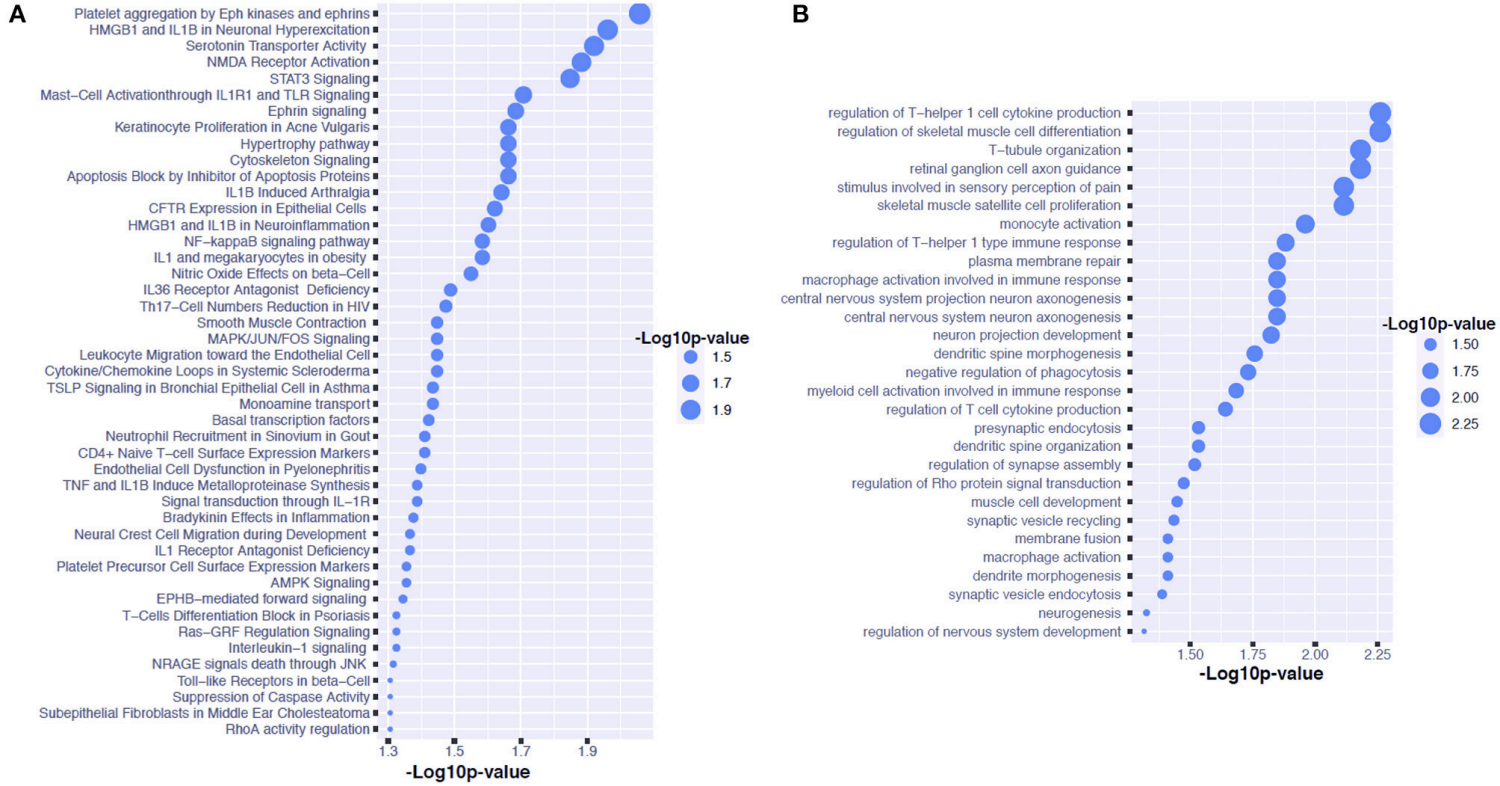
Pathway analysis for candidate genes associated with sleep quality in children on the autism spectrum. **(A)** Shows the GO pathways over-represented by the genes associated with the CSHQ score. **(B)** Shows the signaling pathways over-represented by the genes associated with the CSHQ score.

**Figure 4:**
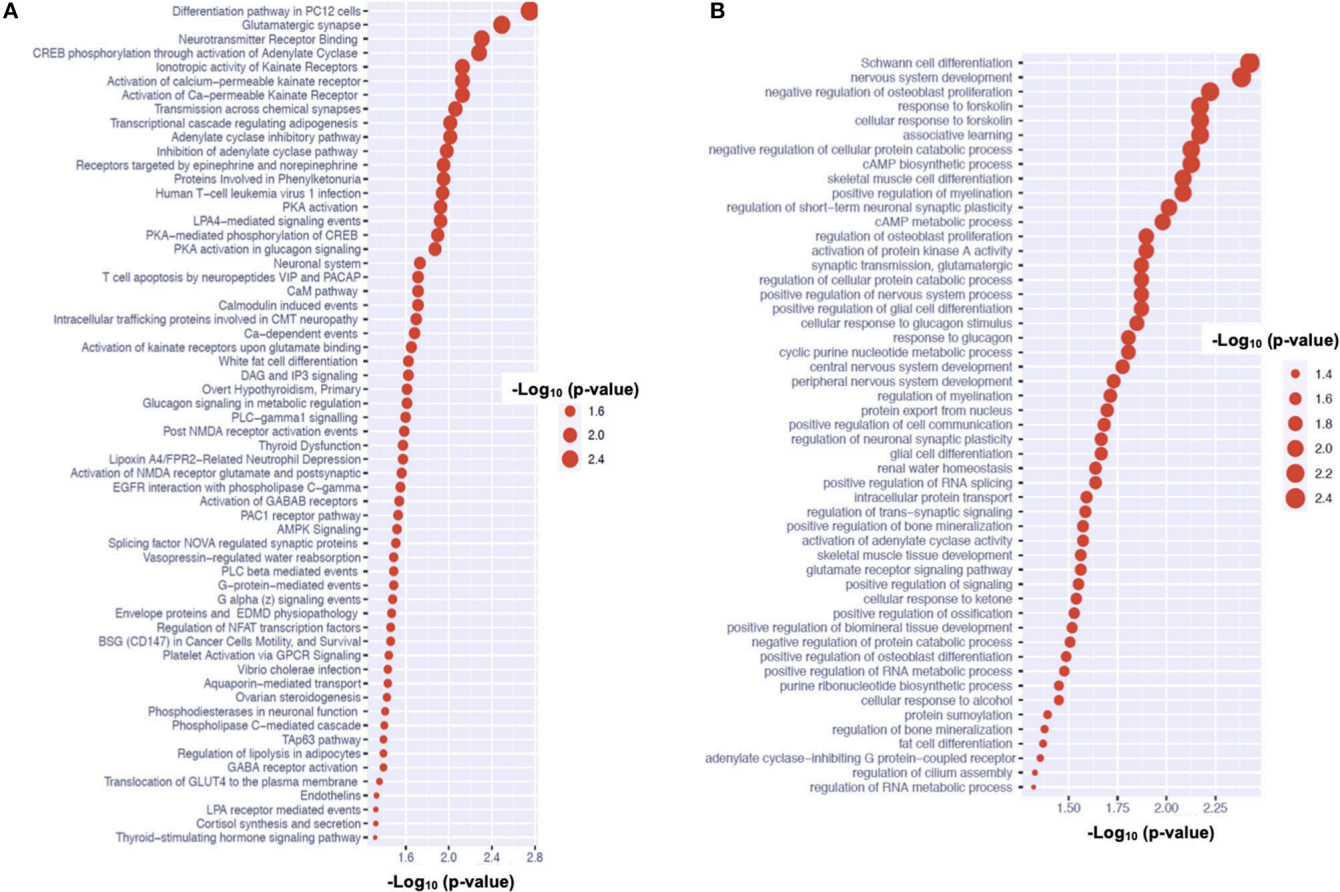
Pathway analysis for candidate genes associated with urine 6-SM levels in children on the autism spectrum. **(A)** Shows the GO pathways over-represented by the genes associated with the 6-SM level. **(B)** Shows the signaling pathways over-represented by the genes associated with the 6-SM level.

We have found 24 and 16 mapped genes for the sleep- and melatonin-associated SNPs, respectively. Although there was no common SNP shared by these two lists of SNPs, there was one common mapped gene: TBC1 Domain Family Member 1 gene (TBC1D1). For the sleep quality, we discovered 45 signaling pathways and 29 GO pathways over-represented by these 24 genes as shown in Figures 3A,B, respectively. The signaling pathway with strongest evidence for enrichment with these genes is the pathway involved in platelet aggregation by Eph kinase and ephrins. The GO pathway with strongest evidence for enrichment with these genes is the pathway involved in regulation of T-helper 1 cell cytokine production. On the other hand, 60 signaling pathways and 51 GO pathways associated with the 6-SM level were found to be over-represented by these 16 genes as shown in Figures 4A,B, respectively. The signaling pathway with strongest evidence for enrichment with these genes is the differentiation pathway in PC12 cells. The GO pathway with strongest evidence for enrichment with these genes is the pathway involved in Schwann cell differentiation.

## Discussion

Our findings indicate that greater 6-SM levels might correlate with poorer sleep quality (i.e., higher CSHQ scores). These findings seem to contradict with the bulk of previous studies that report an inverse relationship between 6-SM levels and the risk of sleep disturbance. Notably, we fuond that the relationship between CSHQ scores and 6-SM levels could vary by age in our sample, in which the positive association between 6-SM levels and CSHQ scores primarily stemmed from the younger children (age: 2–6 years). The relationship between 6-SM levels and sleep quality appeared to be a dome-shaped curve for the adolescents (aged 13–17 years), and hence the assumption of a linear relationship regardless of the age group might need to be scruitinized. An earlier study found that 6-SM levels collected during the daytime might correlate with sleep quality in different ways from nighttime levels (20). The authors inferred that anxiety increases oxidative stress, which in turn causes an increased production of 6-SM because of its antioxidant action. Anxiety may lead to poorer sleep quality and vice versa, which can in turn trigger melatonin secretion. However, our samples lacked data on anxiety symptoms to validate the connection between anxiety and 6-SM levels. Melatonin may play a smaller role in sleep disturbance in children on the autism spectrum compared with neurotypical children. Taken together, these observations may account for the elusive or paradoxical relationship between melatonin levels and sleep quality.

Our first GWAS reveals that the variants of the STON1 gene may be associated with the variation in sleep quality indicated by the CSHQ score amongst individuals on the autism spectrum. The STON1 gene is involved in endocytic machinery, which plays a key role in synaptic functions (21). It is also highly expressed in the cerebellum, pons, and medulla regions. Although the variants of the STON1 gene have not been found to contribute to any behavioral or emotional traits, this gene is involved in the balance between T helper 1 (Th1) and T helper 2 (Th2) cytokine activity toward Th1 dominance, which influences regular nocturnal sleep (22). The signaling pathway analysis results reveal that T-helper 1 cell cytokine production may play a role in sleep disturbances of children on the autism spectrum. Previous evidence suggests that the production of pro-inflammatory cytokines by T1 cells could reach peaks during early nocturnal sleep (23). Our data also suggest that the pathway involved in platelet aggregation by Eph kinase and ephrins. This finding may lend some support to the previous evidence from a mouse model that discloses the role of Eph kinase in rapid eye movement sleep (24).

Our second GWAS indicates that one SNP near the SNAP25 gene may be associated with the 6-SM level. Disruptions of the SNAP25 gene could cause dysregulated circadian rhythm in the schizophrenia-related mouse model (25, 26). Alternative splicing in the SNAP25 gene could also affect the circadian clock in mice (27). The signaling pathway analysis results suggest that PC12 cells, a type of catecholamine cells that synthesize, store and release norepinephrine and dopamine, may be involved in 6-SM levels of children on the autism spectrum. Accumulating evidence reveals that melatonin can inhibit PC12 cell growth (28–30). The GO pathway analysis results highlight the link between Schwann cells and melatonin, which may align with the findings that melatonin can promote Schwann cell proliferations (31–33). The SNAP-25 gene has been associated with distinct brain diseases, including attention deficit hyperactivity disorder (ADHD), schizophrenia and bipolar disorder—which is possibly due to its encoded protein is involved in synaptic functions. The top pathways associated with melatonin metabolism (indicated by 6-SM level) are the pathways involved in Schwanna cell differentiation due to the role of Early Growth Response 2 (EGR2) gene—which is involved in nervous system development (34). We are aware that single-locus association findings may not always align with multi-locus association findings. Pathway-based association findings reflect multi-locus effects, which may stem from joint effects of multiple loci with moderate individual effects.

The only gene found to be associated with both sleep disturbance and 6-SM levels is the TBC1D1 gene, which is involved in the PI3k/Akt signaling pathway. This pathway, which is an intracellular signal transduction pathway that promotes metabolism, proliferation, cell survival, growth and angiogenesis in response to extracellular signals. PI3k/Akt signaling pathway can react to sleep deprivation in animal models (35), which may explain its role in these two phenotypes.

To further clarify whether the genes involved in melatonin metabolism contribute to the variation in 6-SM levels of children on the spectrum, we scruitinized the SNPs in the genes including Arylalkylamine N-Acetyltransferase (AANAT), Tryptophan 2,3-Dioxygenase (TDO2), Cytochrome P450 Family 2 Subfamily C Member 19 (CYP2C19), Cytochrome P450 Family 1 Subfamily A Member 1 (CYP1A1), Tryptophan Hydroxylase 2 (TPH2), and Acetylserotonin O-Methyltransferase (ASMT). The results indicate that none of the SNPs in these genes were significantly associated with the 6-SM level using *p* < 0.0005 (adjusted for multiple tests by taking 100 SNPs into account) as the cutoff. Therefore, the current study suggests that the polymorphisms in these melatonin metabolism-related genes might not contribute substantially to the variation in the melatonin level in children on the spectrum. Other clinical features (e.g., emotional symptoms associated with sleep disturbance) and physiological phenomena (e.g., pro-inflammatory responses) in children on the autism spectrum may also jointly influence the variation in melatonin levels.

The major limitation of our genetic association studies is the relatively small sample size compared with most of the recent large-scale GWAS. A small sample size might be sensitive to clinical heterogeneity that predisposes to inconsistency across different studies because of difficulties in replicating results. Therefore, caution needs to be exercised particularly for our findings on melatonin based on 219 subjects, which contradict with findings from several previous studies. However, the strength of the current study is the combination of sleep-related phenotypic, genetic, and melatonin data, which can allow us to explore the underpinnings of sleep in autism from different persepctives.

Another limitation is the lack of data on 6-SM levels in individuals without autism spectrum disorder because the the current study is aimed to understand biological factors associated with sleep problems in children on the autism spectrum. Therefore, we could not compare the relationship between sleep disturbance and urine 6-SM levels in children on the autism spectrum vs. controls. Furthermore, the genes we found to be associated with either sleep problems or 6-SM levels in our sample only represent candidate genetic factors that contribute to the variation in these two phenotypes within individuals on the autism spectrum.

## Conclusions

Our data suggest that the relationship between melatonin and sleep quality in children on the autism spectrum may be complex since it depends on other factors such as age. Although our GWAS findings do not provide strong or robust evidence for the associations between common variants and either sleep quality or melatonin levels in children on the autism spectrum, our data do suggest potential roles of some genetic networks in either sleep quality or melatonin levels in children on the autism spectrum. GWAS studies using larger and independent samples to replicate these findings are warranted to clarify the relationship between genetic networks, melatonin levels and sleep disturbances, among individuals on the autism spectrum.

## Data Availability Statement

The original contributions presented in the study are included in the article/supplementary material, further inquiries can be directed to the corresponding author.

## Ethics Statement

The studies involving human participants were reviewed and approved by University of New South Wales HREC. Written informed consent to participate in this study was provided by the participants' legal guardian/next of kin.

## Author Contributions

VE and P-IL conceptualized the study. VE and AM contributed to subsequent amendments to the study protocol. P-IL and MM performed the statistical analyses. P-IL wrote the first draft of this manuscript. All authors contributed to subsequent drafts and approved the final manuscript.

## Funding

The authors acknowledge the financial support of the Cooperative Research Center for Living with Autism (Autism CRC), established and supported under the Australian Government's Cooperative Research Centers Program. The funder did not have any role in the design of the study and collection of data, nor in the writing of the manuscript.

## Conflict of Interest

The authors declare that the research was conducted in the absence of any commercial or financial relationships that could be construed as a potential conflict of interest.

## Publisher's Note

All claims expressed in this article are solely those of the authors and do not necessarily represent those of their affiliated organizations, or those of the publisher, the editors and the reviewers. Any product that may be evaluated in this article, or claim that may be made by its manufacturer, is not guaranteed or endorsed by the publisher.
